# Surgical Outcomes for Vertebral Body Hemangiomas With Compressive Symptoms: An Institutional Experience

**DOI:** 10.7759/cureus.73014

**Published:** 2024-11-04

**Authors:** Niraj Kanaujia, Brajesh Kumar, Om Prakash Gupta, Samendra Kumar Singh, Brajesh Kumar

**Affiliations:** 1 Neurosurgery, Indira Gandhi Institute of Medical Sciences, Patna, IND

**Keywords:** absolute alcohol, compressive myelopathy, computed tomography, magnetic resonance imaging, vertebral hemangioma

## Abstract

Background

Vertebral hemangiomas (VHs) are benign vascular tumors commonly found within the vertebral bodies of the spine. While most VHs remain asymptomatic and are often discovered incidentally during imaging studies, a subset can become symptomatic, leading to clinical challenges. The optimal treatment approach for symptomatic VHs remains a topic of debate. At our institution, we have managed aggressive VHs through a combination of surgical decompression and intraoperative alcohol ablation. The objective of this research is to evaluate the effectiveness of a combined treatment approach involving intralesional ethanol injection, surgical decompression, and stabilization using titanium polyaxial screws and rods in managing symptomatic VHs with compressive symptoms.

Methodology

A prospective longitudinal study was conducted at the Indira Gandhi Institute of Medical Science, Patna, in patients with symptomatic VHs associated with compressive myelopathy. The operative plan involved bilateral transpedicular intralesional injection of absolute alcohol (<1% hydrated ethyl alcohol), followed by laminectomy decompression and stabilization of the affected segment using polyaxial titanium pedicle screws and rods.

Results

A total of 19 patients were included in the study, consisting of 10 females and nine males, all presenting with back pain, myelopathy, and sphincter dysfunction. Preoperative American Spinal Injury Association (ASIA) scores were as follows: A (9), B (5), C (3), D (2), and E (0). The average duration of the surgery was 120 minutes, with a standard deviation of 30 minutes. The average blood loss recorded was 250 cc, with a variability of 50 cc, and the typical volume of absolute alcohol utilized was 6 ml, with a standard deviation of 5 ml. Postoperatively, all patients showed improvement, with follow-up ASIA scores of E (9), D (5), B (3), and C (2).

Conclusions

The use of intraoperative ethanol as an embolizing agent, laminectomy decompression, and stabilization using screws and rods for symptomatic VHs seems to be ideal treatment modality in resource-poor countries like India.

## Introduction

Vertebral hemangiomas (VHs) are one of the most common benign lesions of the vertebra. Although normally asymptomatic, in symptomatic cases, they can be quite challenging to manage, occurring in around 0.9-1.2% [1]. The incidence of VH in the general population is about 10% [1]. The most common localization is the dorsal spine, and VH is more frequently observed in females. Most commonly, it occurs at the single level of vertebrae, and involvement of two vertebrae is rare, with involvement of more than two levels being extremely rare [2]. Various treatment approaches have been suggested in the existing literature, including embolization combined with total vertebrectomy, the injection of various "cement" substances, and radiotherapy [3]. Although this procedure of complete vertebrectomy can completely excise the tumor, it has great potential for blood loss, due to which it has high morbidity and mortality. Embolization agents and cement-like substances can only temporarily reduce vascularity and carry the risk of recurrence. While percutaneous ethanol embolization has proven effective, it is associated with a significant risk of pathological fractures [4,5].

Prior studies have mitigated some of these concerns by demonstrating the safety, effectiveness, and practicality of intraoperative ethanol embolization, followed by short-segment fixation using screws and rods [4,6]. This study aims to further investigate the potential role of intraoperative ethanol embolization in combination with surgical decompression and stabilization by the use of titanium polyaxial screws and rods in the management of symptomatic VHs about neurological improvement and in terms of safety.

## Materials and methods

Study design and setting

A prospective longitudinal study was conducted at the Indira Gandhi Institute of Medical Sciences, Patna, involving patients with symptomatic VHs associated with compressive myelopathy. The study commenced after receiving approval from the Institutional Ethics Committee of Indira Gandhi Institute of Medical Sciences (approval number: IEC/IGIMS/2023/12). The study was conducted between January 2024 and April 2024.

Selection Criteria

The selection criteria included patients presenting with symptoms of cord compression and myelopathic features. Each patient underwent a thorough clinical examination and imaging, including plain roentgenograms, CT scans, and contrast-enhanced MRIs. VHs were identified based on characteristic imaging findings: hyperintense signals on T1- and T2-weighted MRI images, with high vascularity on contrast-enhanced imaging, and coarse trabeculae with osseous expansion visible on CT scans. Only patients meeting these diagnostic thresholds were included. Patients with pathological fractures, spinal deformities, or immunosuppressive conditions were excluded.

Data sources and variables

All patients meeting the inclusion criteria underwent pre-anesthetic clearance (PAC) to assess surgical fitness, which included a full blood count, electrolytes, coagulation profile, and cardiovascular evaluations, such as ECG and echocardiography. Patients presenting with abnormalities were reassessed as necessary. Upon receiving PAC clearance, patients underwent surgery by a single surgeon using the same technique. The procedure involved bilateral transpedicular intralesional injection of absolute ethanol (<1% hydrated ethyl alcohol), followed by laminectomy, decompression, and fixation using pedicle screws and rods.

Surgical Steps

Under general anesthesia, patients were positioned prone with the C-arm precisely adjusted for accurate vertebral level identification. A midline incision was made over the spinous process, exposing one vertebral level above and below the affected vertebra. Bilateral pedicle screws (6.5 mm diameter, 40-50 mm length) were inserted into adjacent healthy vertebrae, one level above and one level below the lesion. Titanium rods (5.5 mm diameter) were used with the screws for stabilization.

Using fluoroscopic guidance, 14-16 gauge Jamshidi needles were inserted into the pedicles of the affected vertebra. Absolute ethanol (<1% hydrated) was injected, beginning with 2-3 cc per side, followed by a wait time of two to three minutes. The needles were repositioned to ensure thorough embolization. Ethanol injection continued during needle withdrawal if initial embolization was insufficient.

The entire procedure was conducted using an operating microscope set at 8-12x magnification, enhancing precision and allowing for clear identification of any potential ethanol leakage. In cases of retrograde leakage, the surgical field was irrigated with normal saline, and simultaneous suction was applied. After embolization, laminectomy and decompression of the hemangiomatous tissue were performed via a posterior approach, with nearly bloodless fields due to prior ethanol embolization.

Data Collection

Surgical data, including operative time, ethanol amount, blood loss, and any significant intraoperative or postoperative events, were recorded. Hemangiomatous tissue excised during surgery was sent for histopathological confirmation. Postoperatively, patients were monitored in the ICU for 24 hours, with vital signs (heart rate, blood pressure, respiratory rate, temperature) recorded every four hours and neurological assessments every six hours. Once stabilized, patients were transferred to the ward with daily neurological and pain assessments using the visual analog scale.

Follow-Up Assessments

Patients were followed up post-discharge, with MRI and CT scans conducted every six months to monitor for recurrence or structural changes. Neurological function was evaluated using the American Spinal Injury Association (ASIA) impairment scale (Table 1) [7] at each follow-up, with a focus on any changes in motor and sensory function relative to preoperative baselines.

**Table 1:** ASIA impairment scale ASIA: American Spinal Injury Association

ASIA impairment scale	Description
A - complete	No motor, no sensory, no sacral sparing
B - incomplete	No motor, sensory only
C - incomplete	50% of muscles LESS than grade 3 (can’t raise arms or legs off bed)
D - incomplete	50% of muscles MORE than grade 3 (can raise arms or legs off bed)
E - normal	Motor and sensory functions are normal

Data analysis

Data analysis was performed using Microsoft Excel (Microsoft Corporation, Redmond, WA, USA) and SPSS Statistics software (IBM Corp. IBM SPSS Statistics for Windows. Armonk, NY: IBM Corp.) to ensure accurate descriptive statistics and assessment of surgical outcomes. Key variables, including mean surgical time, intraoperative blood loss, and the volume of alcohol used for embolization, were analyzed. Clinical features such as myelopathy, paraplegia, and back pain were also evaluated. Results were presented in tables, highlighting preoperative and postoperative conditions to illustrate the effectiveness of the treatment approach.

## Results

Table 2 shows the demographics, clinical symptoms, and surgical outcomes of 19 patients with symptomatic VHs. The age range of the patients was 30-64 years, with 10 females and nine males. Clinical symptoms included myelopathy in all patients, nine of whom were paraplegic, with sphincter involvement in two patients. Lower back or mid-back pain was present in 18 patients. Preoperative ASIA scores revealed that nine patients were classified as Grade A, 5 as Grade B, 3 as Grade C, and 2 as Grade D. Eighteen patients had single vertebral involvement, while one patient had multiple vertebral levels involved. All patients underwent posterior decompression and fixation, and post-operative ASIA scores showed significant improvements.

**Table 2:** Patient demographics, symptoms, and surgical outcomes in symptomatic VHs LBA: lower back ache, ASIA: American Spinal Injury Association, VHs: vertebral hemangiomas

Sr. no	Age/sex	Symptoms	ASIA score (preoperative)	Level	Soft tissue compression	Surgery	Postoperative (ASIA score)	Complication
1	62/F	LBA, paraplegic	A	D4	Yes	Posterior decompression and fixation	E	None
2	55/M	LBA, paraplegic	A	D6	Yes	Posterior decompression and fixation	D	None
3	48/M	Mid and LBA, paraplegic	A	D3	Yes	Posterior decompression and fixation	B	None
4	48/M	Mid and LBA	A	D7	Yes	Posterior decompression and fixation	E	None
5	59/F	LBA	B	D9	Yes	Posterior decompression and fixation	E	None
6	49/F	LBA	B	D11	Yes	Posterior decompression and fixation	E	None
7	42/M	LBA, paraplegic, sphincter dysfunction	B	D3	Yes	Posterior decompression and fixation	C	None
8	49/F	LBA, paraplegic	A	D6	Yes	Posterior decompression and fixation	D	None
9	63/F	Mid and LBA	C	D5	Yes	Posterior decompression and fixation	E	None
10	62/F	LBA, paraplegic	A	D4	Yes	Posterior decompression and fixation	C	None
11	55/M	LBA, paraplegic	A	D6	Yes	Posterior decompression and fixation	B	None
12	48/M	Mid and LBA, paraplegic	A	D3	Yes	Posterior decompression and fixation	E	None
13	48/M	Mid and LBA	D	D7	Yes	Posterior decompression and fixation	E	None
14	59/F	LBA	B	D9	Yes	Posterior decompression and fixation	E	None
15	49/F	LBA	B	D11	Yes	Posterior decompression and fixation	D	None
16	42/M	LBA, paraplegic, sphincter dysfunction	B	D3	Yes	Posterior decompression and fixation	B	None
17	49/F	LBA, paraplegic	A	D6	Yes	Posterior decompression and fixation	E	None
18	63/M	Mid and LBA	C	D5, D6	Yes	Posterior decompression and fixation	D	None
19	62/F	LBA, paraparesis	C	D4	Yes	Posterior decompression and fixation	D	None

Table 3 outlines the clinical and surgical characteristics of the study population. The average duration of the surgery was 120 ± 30 minutes, with an average blood loss of 250 ± 50 cc. The typical volume of absolute alcohol utilized for embolization was 6 ± 5 ml. Postoperatively, all patients showed improvement at the six-month follow-up, with follow-up ASIA scores indicating nine patients reached Grade E, five patients reached Grade D, three were Grade B, and two were Grade C, showing overall positive surgical outcomes.

**Table 3:** Clinical and surgical characteristics of the study population ASIA: American Spinal Injury Association

Characteristic	Value
Total number of patients	19
Age range (years)	30–64
Female patients	10
Male patients	9
Myelopathy	19
Paraplegic	9
Sphincter involvement	2
Mid-to-lower back pain	18
Preoperative ASIA scores - grade A	9
Preoperative ASIA scores - grade B	5
Preoperative ASIA scores - grade C	3
Preoperative ASIA scores - grade D	2
Preoperative ASIA scores - grade E	0
Single vertebral involvement	18
Multiple vertebral involvement	1
Mean surgical time (minutes)	120 ± 30
Mean blood loss (cc)	250 ± 50
Mean amount of absolute alcohol used (ml)	6 ± 5
Postoperative improvement (6-month follow-up)	All patients improved
Follow-up ASIA scores - grade E	9
Follow-up ASIA scores - grade D	5
Follow-up ASIA scores - grade B	3
Follow-up ASIA scores - grade C	2

## Discussion

VHs are primarily benign vascular tumors that can lead to significant morbidity when they compress the spinal cord or nerve roots. This study examined a cohort of 19 patients with symptomatic VHs, offering critical insights into their clinical features, surgical management, and outcomes. All patients presented with clinical features of myelopathy, highlighting the aggressive nature of these lesions. Mid- or lower-back pain was the most common symptom, reported by 18 out of 19 patients, while neurological deficits, including paraplegia and sphincter involvement, were present in a significant number. These findings are consistent with previous literature, where back pain is a predominant symptom in up to 54% of cases with symptomatic VHs [5].

The preoperative ASIA scores indicated substantial neurological impairment, with Grade A (complete loss of motor and sensory function) observed in nine patients. This finding aligns with reports that aggressive VHs can cause severe neurological deficits [6]. In this cohort, surgical intervention was determined based on the extent of vertebral involvement; most patients had a single vertebral lesion, which is consistent with prior findings that solitary lesions are more common in symptomatic cases [6]. Surgical management followed a consistent technique of bilateral transpedicular intralesional absolute alcohol injection, laminectomy, and decompression. The average surgical time was 120 ± 30 minutes, with an average blood loss of 250 ± 50 cc, demonstrating the efficacy of preoperative alcohol embolization in reducing intraoperative bleeding, a finding supported by previous studies [7,8]. This technique not only facilitated decompression but also induced sclerotic changes within the hemangioma, corroborating findings from a study that reported a high success rate with this approach [7].

Postoperative follow-up at six months showed significant improvement in ASIA scores, with nine patients improving to Grade E and 5 to Grade D. These results are consistent with literature reporting favorable outcomes following alcohol ablation in VH treatment [8]. However, the injection volume of alcohol must be carefully controlled, as excessive amounts can compromise vertebral integrity, leading to pathological fractures [9].

The debate over the role of surgical intervention in VH continues, with procedure choices ranging from laminectomy to vertebrectomy, often based on the degree of neural compression. In this cohort, decompression was the primary goal, aligning with current treatment trends favoring conservative surgical techniques that maintain effectiveness [10-12]. Postoperative care included consideration for adjunctive therapies, such as endovascular techniques and radiotherapy, particularly for patients with incomplete resection or requiring pain management. Transarterial embolization is a recognized option for these patients, with documented safety and efficacy [13]. Additionally, low-dose radiation therapy, once more widely used in cases of incomplete resection, is now less commonly applied due to advances in other adjuvant therapies [14]. These multimodal approaches facilitate tailored treatment strategies optimized for individual patient factors and improve overall VH management outcomes [15].

Strengths and limitations

This study’s strengths include the consistent surgical technique employed across cases, which enhances the reliability of the findings. The prospective longitudinal design offers a clearer assessment of treatment outcomes over time. Furthermore, the use of standardized assessment tools, such as the ASIA impairment scale, for evaluating neurological improvement adds a layer of objectivity to the data collection process.

However, the study has several limitations. The small sample size of 19 patients may restrict the generalizability of the findings, as larger cohorts would provide more comprehensive data on treatment effectiveness and outcome variability. Additionally, the absence of a control group limits direct comparisons between surgical and conservative management outcomes. The six-month follow-up, while providing insight into initial outcomes, may not fully capture long-term recurrence or complications. Variations in individual characteristics, such as age, comorbidities, and surgical nuances, may introduce biases affecting the interpretation of the efficacy and safety of the interventions used.

Future research directions

Future studies with larger, multicenter cohorts and extended follow-up durations are needed to validate these findings and to establish standardized treatment protocols for VHs. Investigating alternative embolization agents or techniques may further reduce risks, such as pathological fractures. Moreover, future research could explore the role of adjuvant therapies, including radiotherapy or endovascular techniques, as complementary treatments to surgical intervention, particularly for aggressive or recurrent VH cases.

In terms of clinical implications, this study highlights the potential of a minimally invasive approach in managing symptomatic VHs, which could be particularly beneficial in resource-limited settings. The integration of intraoperative ethanol embolization with decompressive laminectomy provides a pragmatic solution for VH management, allowing treatment to be adapted based on the availability of resources and specific patient needs. Additionally, further research could assess combining this approach with other modalities, such as radiotherapy or endovascular techniques, to enhance overall outcomes.

## Conclusions

The findings of this study underscore the importance of timely surgical intervention in patients with symptomatic VHs, particularly when accompanied by preoperative alcohol embolization. The significant improvement in ASIA scores postoperatively illustrates the potential for successful recovery in these patients. Despite the limitations inherent in the study, the results contribute valuable insights into the management of aggressive VHs, supporting the notion that a multimodal approach tailored to individual patient needs can lead to favorable outcomes. Further research with larger cohorts and longer follow-up periods is essential to establish standardized treatment protocols and improve the management of VHs.
